# Academic Life in Emergency Medicine (ALiEM) Blog and Podcast Watch: Infectious Disease Emergencies

**DOI:** 10.7759/cureus.2345

**Published:** 2018-03-19

**Authors:** Lynn Roppolo, Chris Gaafary, Jay Khadpe, Kaushal Shah, Andrew Grock

**Affiliations:** 1 Department of Emergency Medicine, University of Texas Southwestern, Dallas, Texas; 2 University of South Carolina College of Medicine Greenville, University of South Carolina College of Medicine Greenville / Greenville Health System, Greenville, Sc; 3 Department of Emergency Medicine, University of Florida College of Medicine, Jacksonville, Florida; 4 Department of Emergency Medicine, Icahn School of Medicine at Mount Sinai Medical Center, New York, Ny; 5 Department of Emergency Medicine, UCLA

**Keywords:** foam, online education, infectious disease, emergency medicine, aliem

## Abstract

To address the needs for curation of online educational content as well as the development of a nationally available curriculum that meets individualized interactive instruction, the Academic Life in Emergency Medicine (ALiEM) Approved Instructional Resources (AIR) Series and AIR-Pro Series were created in 2014 and 2015, respectively. Using an expert-based, crowd-sourced approach, these two programs identify trustworthy, high-quality, educational blog, and podcast content.

Here, we summarize the accredited posts that met our a priori determined quality criteria and evaluated by eight attending physicians.

## Introduction and background

Despite the rapid rise of educational content available through blogs and podcasts in emergency medicine (EM), [1] identification of quality resources for educators and learners has only made preliminary progress [2-4]. In 2008, the Accreditation Council for Graduate Medical Education endorsed a decrease in synchronous conference experiences for EM residency programs by upto 20% in exchange for asynchronous learning termed Individualized Interactive Instruction (III) [5].

To address this need, the Academic Life in Emergency Medicine (ALiEM) Approved Instructional Resources (AIR) Series and AIR-Pro Series were created in 2014 and 2015, respectively, to help EM residency programs identify quality online content on social media [6-7]. Using an expert-based, crowd-sourced approach, these two programs identify trustworthy, high-quality, educational blog and podcast content. For the ALiEM Blog and Podcast Watch, summaries of these posts are written by the AIR and AIR-Pro Series’ editorial boards [8-9]. This installment from the AIR Series summarizes the highest scoring social media educational resources on infectious disease emergencies.

## Review

Topic identification

The Approved Instructional Resources (AIR) Series is a continuously building curriculum based on the Council of Emergency Medicine Residency Directors (CORD) testing schedule [10].

Inclusion and Exclusion Criteria

A search of the 50 most frequently visited sites per the Social Media Index [11] was conducted for resources relevant to infectious disease emergencies, published within the previous 12 months. The search, conducted in August 2016 included blog posts and podcasts written in English for our scoring by our expert panel.

Scoring

Extracted posts were scored without blinding by eight reviewers from the AIR Editorial Board, which is comprised of EM core faculty from various United States medical institutions. The scoring instrument contains five measurement outcomes using seven-point Likert scales: Best Evidence in Emergency Medicine (BEEM) score, accuracy, educational utility, evidence-based, and references (Table 1) [12]. More detailed methods are described in the original description of the AIR Series [6-7]. Board members with any role in the production of a reviewed resource recused him/herself from grading that resource.

**Table 1:** Approved Instructional Resources scoring instrument for blog and podcast content with the maximum score being 35 points. (BEEM = Best Evidence in Emergency Medicine; EP = emergency physician; EBM = Evidence Based Medicine)

Tier 1: BEEM Rater Scale	Score	Tier 2: Content accuracy	Score	Tier 3: Educational Utility	Score	Tier 4: Evidence Based Medicine	Score	Tier 5: Referenced	Score
Assuming that the results of this article are valid, how much does this article impact on EM clinical practice?		Do you have any concerns about the accuracy of the data presented or conclusions of this article?		Are there useful educational pearls in this article for senior residents?		Does this article reflect evidence based medicine (EBM)?		Are the authors and literature clearly cited?	
Useless information	1	Yes, many concerns from many inaccuracies	1	Not required knowledge for a competent EP	1	Not EBM based, only expert opinion	1	No	1
Not really interesting, not really new, changes nothing	2		2		2		2		2
Interesting and new, but doesn't change practice	3	Yes, a major concern about few inaccuracies	3	Yes, but there are only a few (1-2) educational pearls that will make the EP a better practitioner to know or multiple (>=3) educational pearls that are interesting or potentially useful, but rarely required or helpful for the daily practice of an EP.	3	Minimally EBM based	3		3
Interesting and new, has the potential to change practice	4		4		4		4	Yes, authors and general references are listed (but no in-line references)	4
New and important: this would probably change practice for some EPs	5	Minimal concerns over minor inaccuracies	5	Yes, there are several (>=3) educational pearls that will make the EP a better practitioner to know, or a few (1-2) every competent EP must know in their practice	5	Mostly EBM based	5		5
New and important: this would change practice for most EPs	6		6		6		6		6
This is a "must know" for EPs	7	No concerns over inaccuracies	7	Yes, there are multiple educational pearls that every competent EP must know in their practice	7	Yes exclusively EBM based	7	Yes, authors and in-line references are provided	7

Resources with a mean evaluator score of ≥ 30 points (out of a maximum of 35) are awarded the AIR label. Resources with a mean score of 27-29 and deemed accurate and educationally valuable by the reviewers are given the Honorable Mention label.

Results

We initially included a total of 85 blog posts and podcasts. Key educational pearls from the two AIR posts and the 18 Honorable Mentions are described.

Article 1. Badari S. Diarrhea, “Answers”. EMLyceum. (November 2nd, 2015) AIR

https://emlyceum.com/2015/11/02/diarrhea-answers/

This post provides a thorough evidence-based review of the Emergency Department (ED) workup and treatment of patients who present with diarrhea. The take-home points are as follows:

Typically, most patients with diarrhea need little work-up in the ED. Those with high-risk features (high fever > 101.3, persistent diarrhea - defined as > seven days, severe abdominal pain, bloody diarrhea, immunocompromised, elderly or systemically ill) may benefit from fecal white blood cells (WBC) and red blood cells (RBC), or fecal occult blood with stool cultures if any of these tests are positive. For patients with diarrhea without high-risk features, no diagnostic work up is indicated and antibiotics are unnecessary. Ciprofloxacin may reduce the duration of symptoms in suspected invasive disease as long as hemolytic-uremic syndrome and thrombotic thrombocytopenic purpura not suspected. Loperamide may be helpful in healthy adults with uncomplicated diarrhea but should not be given to pediatric patients. Probiotics, specifically Lactobacillus, have shown promise for prevention and treatment of infectious and antibiotic-associated diarrhea.  

Article 2. Farkas, J. PulmCrit—Neurocritical care of the comatose meningitis patient. EMCrit. (December 2015) (http://emcrit.org/pulmcrit/neurocritical-care-meningitis-icp/)

This post reviews six key management steps to improve outcomes in severe meningitis, covering the following take-home points:

In bacterial meningitis, the six key steps are: early corticosteroid administration, cerebrospinal fluid drainage if opening pressures are greater than 27 mmHg on lumbar puncture, close temperature monitoring with antipyretics to avoid fever, anticonvulsants for seizures, and electroencephalogram to evaluate for nonconvulsive status, as well as considering imaging to evaluate for sinus vein thrombosis, infarction, subdural empyema, or brain abscess. It should be kept in mind that cerebral perfusion pressure could be low in cases of elevated ICP and borderline mean arterial pressure. Norepinephrine may be necessary to ensure a cerebral perfusion pressure > 60mmHg.

Article 3. Farkas, J. Evidence-based treatment for severe community-acquired pneumonia. EMCrit. (August 16, 2015) HM

http://emcrit.org/pulmcrit/evidence-based-treatment-for-severe-community-acquired-pneumonia/

The blog post reviews the evidence for severe community-acquired pneumonia (CAP) treatments including antibiotics and steroids. The take-home points are as follows:

This article recommends both a beta-lactam and azithromycin for severe CAP over fluoroquinolones. The evidence for steroids, on the other hand, in cases of severe CAP is less clear. Steroids have shown reduce hospital length of stay and may reduce the need for intubation, but should be avoided in patients with contraindications, fluoroquinolone treatment, and possible influenza infection.

Article 4. Farkas, J. PulmCrit: Which patients admitted for pneumonia need MRSA coverage? EMCrit. (July 18, 2016) HM

http://emcrit.org/pulmcrit/pneumonia-mrsa/http://emcrit.org/pulmcrit/pneumonia-mrsa/

This post reviews the literature and proposes an algorithm to guide who should and should not be empirically treated for methicillin-resistant staphylococcus aureus (MRSA) pneumonia. The following take-home points are covered:

Physicians are of course concerned with appropriate broad-spectrum antibiotic use. Overuse can contribute to environmental resistance while underuse can result in undertreated bacterial infections. The provided algorithmic approach seeks to balance these risks and benefits to determine MRSA pneumonia using the Shorr score. The four criteria are: (1) age < 30 or > 79, (2) prior healthcare exposure, (3) severity of illness and (4) comorbid illness. If it is low (0–1) in the absence of clinically obvious MRSA, MRSA coverage is unnecessary. If the score is high (6–10), it is reasonable to start empiric coverage for MRSA. MRSA coverage in moderate Shorr scores (2–5) depends on each patient. Aggressive de-escalation of MRSA coverage is also important, though less applicable to EM physicians.

Article 5. Morgenstern J. Articles of the month Special Edition: Pediatric UTI. First10EM. (February 2, 2016) HM

https://first10em.com/2016/02/02/pediatric-uti/

This post reviews the literature regarding pediatric urinary tract infections (UTIs) through several clinically relevant scenarios. The take-home points are as follows:

Diagnosing a simple UTI actually contains considerable challenges. In asymptomatic and nontoxic patients, a positive urinalysis or even urine culture should not be treated. Untreated UTIs rarely lead to pediatric sepsis, and the feared renal scarring on imaging does not correlate to long-term renal function or blood pressure. Imaging is not routinely necessary in children diagnosed with a UTI unless otherwise indicated based on history and physical. 

Article 6. Helman, A. Episode 68 Emergency Management of Sickle Cell Disease. Emergency Medicine Cases (August 2015) HM

http://emergencymedicinecases.com/emergency-management-of-sickle-cell-disease/

This blog post reviews presentation, diagnosis, and treatment of various sickle cell disease (SCD) complications. The take-home points are as follows:

SCD complications reviewed include pain crisis, acute chest syndrome, sepsis, stroke, and eye trauma. Pain crisis is a clinical diagnosis since vital signs and laboratory tests can be normal. Treatment includes aggressive opioids, with an initial IV dose equal to the patient’s usual total daily dose. If no IV access is possible, the subcutaneous route is more reliable than the intramuscular route. Nonsteroidal anti-inflammatory drugs (NSAIDS) may help but should be used sparingly secondary due to the higher rate of renal infarcts in SCD patients. IV fluid boluses and supplemental oxygen are not indicated and can cause harm. Supplemental oxygen can contribute to bone marrow suppression and increased transfusion requirements. Likewise, fluid boluses can increase the risk of acute chest as well as promote sickling by causing hyperchloremic metabolic acidosis. Supplemental oxygen is only indicated if oxygen saturation is < 92% and IV fluids only with clinical hypovolemia.

As SCD patients are asplenic, they are at greater risk for infections. Any fever should be concerning for bacterial infection. Acute chest syndrome should be presumed in any SCD patient with hypoxia or new infiltrate on chest X-ray, and the patient should be admitted. In addition, SCD patients are at greater risk for stroke, for which exchange transfusion, rather than thrombolysis, is the preferred therapy. Lastly, SCD patients are at high risk for hyphemas and traumatic glaucoma, even after mild eye trauma. A slit lamp examination and intraocular pressures are typically indicated.

Article 7. Fox S. UTI Empiric Antibiotics. Peds EM Morsels. (April 15, 2016) HM

http://pedemmorsels.com/uti-empiric-antibiotics/ This blog post provides evidence-based recommendations for diagnosis and management of UTIs in pediatric patients. The take-home points are as follows:

Pediatric UTI empiric therapy should be guided by local antibiotic resistance patterns. Across all age groups, E. coli is the most common pathogen (80%), but infants and neonates are particularly susceptible to Group B streptococcus and enterococcus. A five-day course of oral antibiotics is effective for simple UTIs, while more complicated cases should be admitted for IV antibiotics. Cephalosporins are the first line of defense, and amoxicillin with clavulanate is the next option given increased resistance to amoxicillin or ampicillin alone. Nitrofurantoin can be used for nitrite negative UTIs without pyelonephritis; ciprofloxacin can be used in special cases like cystic fibrosis. Trimethoprim/sulfamethoxazole (TMP/SMX) should not be used empirically. Lastly, seemingly healthy children with abnormal urinalysis may not benefit from empiric therapy. A urine culture should be sent and outpatient follow up confirmed.

Article 8. Fox, S. Sickle Cell Disease and Fever. Peds EM Morsels. (March 18, 2016) HM

http://pedemmorsels.com/sickle-cell-disease-fever/

This blog discusses evaluation and management of SCD patients with fever as well as with serious bacterial infections. The take- home points are as follows: 

Sepsis remains the most common cause of mortality in SCD patients; pneumonia, the most common source for this sepsis. Thus, SCD patients with a fever of 38.5°C (101.3°F) require a septic work up including a complete blood count with differential, reticulocyte count, blood culture, urine culture if UTI is suspected and a chest X-ray if the patient has respiratory symptoms or an abnormal lung examination. Empiric antibiotics such as ceftriaxone should be given that cover S. pneumonia and gram-negative enteric organisms. Though SCD patients with fever typically require admission, outpatient management has been proposed in low-risk patients: >12 months old, well appearing, normal vital signs, no ceftriaxone within the past eight weeks, up to date on vaccinations, no history of severe illness (e.g. sepsis), normal ED workup, and no compliance concerns. It is imperative to discuss the patient’s disposition with their primary hematologist prior to discharge and arrange close follow-up. 

Article 9. Pike L and Villar J. Zika Virus: What Emergency Department Providers Need to Know. Academic Life in Emergency Medicine. (February 15, 2016) HM

https://www.aliem.com/2016/zika-virus-what-emergency-department-providers-need-to-know/

This blog provides a concise overview of the outbreak, clinical presentation, risk factors, diagnostic testing and management of the Zika virus. The following take home points are discussed:

Zika virus gained worldwide attention when the epidemic of associated micocephaly was reported in Brazil in 2015.Though primarily transmitted through Aedes mosquito bites, which also transmit dengue and chikungunya, perinatal transmission is also possible. Additionally, transmission through sexual intercourse and blood transfusion have been reported. Symptoms include fever, rash, joint pain, and conjunctivitis typically beginning two to seven days after transmission. Importantly, most people with Zika are asymptomatic. One should avoid nonsteroidal anti-inflammatory drugs (NSAIDs) until dengue is excluded to avoid risk of hemorrhage. At the time of publishing, the following areas are at risk: Cape Verde in Africa, several areas in the Caribbean, Central America, Mexico and South America, Fiji, Somoa, and Tunga. Zika virus testing is only performed at the Center for Disease Control (CDC) and is reserved for the following special patient populations: acute onset of illness and have traveled to a high risk area within the past two weeks, pregnant women who have traveled to a high risk area within two to 12 weeks, infants with microcephaly or intracranial calcifications born to women who traveled to a high risk area, or infants with mothers with positive or inconclusive test results for Zika virus infection.

*Article 10. Sobolewski, B* **.** *The Febrile Infant – University of Cincinnati Emergency Medicine Collaboration. PEM Cincinatti. (March 1, 2016) HM*

http://pemcincinnati.com/blog/the-febrile-infant-taming-the-sru-collaboration/

This blog post provides recommendations for diagnosis and management of febrile infants without a source between four weeks and 60 days old based on a detailed review of current literature and the opinions of two experts in this field. The following take home points are discussed:

Prevalence of serious bacterial infections (SBI) in febrile infants without a source is much lower in infants four to 12 weeks old compared to those younger than four weeks old. UTI is the most common source, while meningitis is rare. Low-risk clinical criteria for meningitis include: 29-60 days old, full term, no chronic medical problems, no prolonged neonatal intensive care unit (ICU) stay, no systemic antibiotics in the previous 72 hours, well appearing and easily consolable, and with no infections on exam. Low risk work-up criteria for meningitis include: WBC ≤ 15,000 or ≥ 5,000, band neutrophil < 0.2, urine WBC < 10/HPF, negative urine gram stain, and a chest x-ray without infiltrate. For low-risk, febrile, 29- to 60-day-old infants, blood and urine studies alone may be appropriate. If heart rate improves after defervescence and close outpatient follow-up is available, these patients may be appropriate for discharge.

Empiric antibiotics should only be given if a lumbar puncture is performed. For 0- to 21-day-old infants, give ampicillin, cefotaxime, and acyclovir; for 22- to 28-day-old infants, ampicillin and cefotaxime; and for 29- to 56-day-old infants, cefotaxime or ceftriaxone. If there is pleocytosis in the cerebrospinal fluid (CSF) or a positive gram stain, consider adding vancomycin for MRSA coverage. Due to ototoxicity, gentamicin has become less commonly used, but can nonetheless be substituted for cefotaxime in the infant < 14 days.

While HSV is highly unlikely outside of three weeks of age, patients with seizure or an abnormal neurologic examination should receive acyclovir, an HSV CSF polymerase chain reaction (PCR), and hepatic profile to investigate for invasive HSV transaminitis. Enterovirus meningitis occurs predominantly from August to October. Any infant less than 60 days old treated with antibiotics after a full septic work-up in this age range should be admitted.

Article 11. Brooks, D. Blood Cultures: When Do They Make a Meaningful Impact on Clinical Care. EM Docs. (April 23, 2016) HM

http://www.emdocs.net/blood-cultures-when-does-obtaining-them-make-a-meaningful-impact-on-clinical-care/

This post reviews the yield of blood cultures and when they might be useful and recommended. The take-home points are:

A number of studies corroborate the very low yield of blood cultures (3%) and an even lower rate of changing patient management. Despite certain core measures and reimbursement plans requiring them, blood cultures are rarely useful in stable, immunocompetent patients with common infections such as cellulitis, pneumonia, and orchitis. However, those who are acutely ill, septic, presumed bacteremia, or suspected to have endocarditis should have blood cultures drawn prior to initiating antibiotics. Interestingly, fever at the time of blood culture collection is not sensitive or specific.

*Article 12. Triplett, L. Spinal Epidural Abscess. Core EM. (December 30, 2015) HM*****

http://coreem.net/core/spinal-epidural-abscess/

This post reviews the challenging clinical evaluation, work-up, and treatment of spinal epidural abscess (SEA). The take home points are:

Though classically presenting with fever, back pain, and neurologic deficit, this classic triad of SEA occurs only in 8%-15% of cases. Fever itself is only present in 50% of SEA patients and predisposing factors in only 20% percent of cases. The WBC is unhelpful at ruling in or ruling out SEA. Though supported by one recent guideline, this post recommends against using erythrocyte sedimentation rate or C-reactive protein in order to test for SEA. Blood cultures may be helpful in tailoring antibiotic therapy. Magnetic resonance imaging (MRI) with gadolinium is the gold standard for diagnosis – though knowing when to get the MRI can be extremely difficult. Treatment includes vancomycin plus a third-generation cephalosporin to cover MRSA and gram negative bacilli. Operative drainage is not mandatory, though is thought to be more helpful in cases with progressing neurologic developments and abscess on MRI, especially cervical and thoracic SEAs.

Article 13. Helman, A. Fever in the Returning Traveler Emergency Medicine Cases. (April 2015) HM

http://emergencymedicinecases.com/fever-returning-traveler/

This post reviews the approach to fever in the returning traveler including presentation, diagnosis, and treatment of common deadly tropical diseases. The take-home points are:

Every febrile ED patient must be asked about recent travel! If yes, immunizations, malaria prophylaxis, high-risk foods, animal/insect exposures and fresh water activities are key questions to ask. Unfortunately, these common deadly tropical diseases often present with vague symptoms. For patients who have traveled to endemic areas, consider workups for malaria, dengue, typhoid and rickettsia. Of concern, malaria can still occur even in the absence of the classic triad, reported chemoprophylaxis, or a negative initial smear. Clinical cues for typhoid fever presentations include salmon-colored rose spots, blanching, maculopapules on the trunk and extremities, as well as pardoxical bradycardia. Dengue fever can present with fever plus rash (“islands of white in a sea of red”), arthralgias, nausea/vomiting, positive tourniquet test (inflate blood pressure cuff for five minutes and look for distal petechiae on deflation), and leukopenia. Dengue hemorrhagic fever presents with shock, massive plasma leak, disseminated intravascular coagulation (DIC), thrombocytopenia, and hemorrhage.

Article 14. Hofmann, E. Lactate in Sepsis: Pearls & Pitfalls. EM Docs. (March 6, 2016) HM

http://www.emdocs.net/8362-2/

This post reviews lactate metabolism and its role in sepsis including using clearance as a marker for resuscitation. The take-home points are:

Most lactate is produced through anaerobic glycolysis. It can be categorized into Type A which is associated with tissue hypoperfusion, and Type B, associated with causes other than impaired tissue oxygenation. For sepsis, there is ample evidence associating hyperlactatemia with mortality. In fact, lactate continues to be the best non-invasive marker for illness severity in sepsis. In a septic patient, a lactate > 4mmol/L suggests the patient may benefit from admission to the intensive care unit. A lactate < 2 mmol/L suggests minimal mortality risk. For patients with a lactate from 2-4 mmol/L, this article recommends re-checking the lactate after 2 L of normal saline. While a >10% decrease, or normalization, is reassuring, a lack of lactate clearance indicates severe disease and once again ICU level care may be indicated.

Article 15. Rezaie S. Timethoprim-Sulfamethoxazole for Uncomplicated Skin Abscesses? (March 10, 2016) HM

http://rebelem.com/trimethoprim-sulfamethoxazole-for-uncomplicated-skin-abscesses/

This post dissects a recent study that investigates the use of trimethoprim-sulfamethoxazole (TMP-SMX) in the treatment of uncomplicated skin abscess status post incision and drainage [13].The take-home points are:

The study was a double-blind, randomized controlled trial of placebo versus TMP/SMX for seven days in over 1200 patients with an abscess with overlying cellulitis. On follow-up, clinical cure was significantly more frequent in the TMP/SMX group (NNT=14, absolute difference 6.9-7.2%), though the clinical significance of this difference in practice remains unclear. No significant side-effects were noted, but increased antibiotic use may promote increased bacterial resistance. Expert opinion by the post’s author suggests a “wait and see approach” in which a prescription is given with instructions to start the antibiotics if no significant improvement was noted after 48 hours.

Article 16. Levesque, A. Updates on Recommendations For STI Treatments & Empiric Therapy: When to Treat and What to Treat Depending on your Patient. EM Docs. (March 3, 2016) HM

http://www.emdocs.net/updates-recommendations-sexually-transmitted-infection-treatments-empiric-therapy-treat-treat-depending-patient/

This post reviews the latest guidelines on sexually transmitted infections (STIs) in the ED. The take-home points are:

Clinically presumed N. gonorrhoeae (NG) and C. trachomatis (CT) can be treated with ceftriaxone 250mg intramuscularly (IM) and azithromycin 1 gram orally in the ED. CT can also be treated with doxycycline 100mg by mouth (PO), twice a day, (BID), for seven days. For NG conjunctivitis or disseminated NG, the ceftriaxone dose should be increased to 1 gm intramuscularly. Trichomonas often concomitantly occurs with NG and CT and presumptive treatment. Metronidazole 2 grams PO once, or 500mg BID for seven days, should be considered as well. For post-exposure prophylaxis, all persons should be offered a morning-after pill, the hepatitis B vaccine (if unvaccinated), and treatment for NG, GC, and trichomonas. HIV prophylaxis should be discussed with the patient as well. The full CDC 2015 guidelines can be found here: https://www.cdc.gov/std/tg2015/2015-wall-chart.pdf [14].

Article 17. Farkas, J. Steroids in Septic Shock: Four Misconceptions and One Truth. EM Crit. (July 12, 2015) HM

http://emcrit.org/pulmcrit/steroids-in-septic-shock-four-misconceptions-and-one-truth/

This post reviews the evidence for and against the use of corticosteroids in sepsis.The take-home points are:

Several myths have been studied regarding steroids in sepsis. Currently, there is no convincing evidence that steroids reduce mortality in patients with sepsis. On the other hand, there is no data that steroids lead to an increased rate of superinfections. Much of the current confusion about steroids in sepsis stems from the two major studies, Annane et al. and the CORTICUS trial. Despite the authors’ differing conclusions, their data is entirely consistent. Both in fact show no change in mortality, faster shock resolution, and no increased rate of superinfections. Overall, it remains unclear which patients will benefit most from steroids and they should be considered on a case by case basis.

Article 18. Fox, S. Bulging Fontanelle. Peds EM Morsels. (March 4, 2016) HM

http://pedemmorsels.com/bulging-fontanelle/ 

This blog post reviews the differential diagnosis and work-up of a bulging fontanelle.The take-home points are:

Of the six fontanelles, only the anterior and posterior are clinically apparent. Normally, the fontanelles should be slightly sunken and palpable pulsations are normal. Fontanelles can be bulging based on false positives such as supine position, coughing, crying, or vomiting. While numerous benign etiologies exist and work-ups are often negative, a bulging fontanelle is a high-risk exam finding concerning for meningitis and brain mass. In extremely well-appearing, afebrile patients, an outpatient MRI with close follow-up may be appropriate. In all other cases, neuro-imaging and a lumbar puncture are recommended.

## Conclusions

The ALiEM Blog and Podcast Watch series serves to identify high-quality educational blogs and podcasts for EM clinicians through its expert panel, using an objective scoring instrument. These social media resources are currently curated in the ALiEM AIR and AIR-Pro Series, originally created to address EM residency needs. The resources curated specifically for infectious diseases are herein shared and summarized to help clinicians filter the rapidly published multitude of blog posts and podcasts. Our search was limited to content produced within the previous 12 months from the top 50 Social Media Index sites. While these lists are by no means a comprehensive analysis of the entire internet for this topic, this series provides a post-publication accreditation and curation of recent online content to identify and recommend high-quality educational social media content for the EM clinician. While this article focuses on infectious diseases, additional AIR modules address other topics in emergency medicine.

## References

[1] Cadogan M, Thoma B, Chan TM (2014 Oct). Free Open Access Meducation (FOAM) the rise of emergency medicine and critical care blogs and podcasts (2002-2013). Emerg Med J.

[2] Paterson QS, Thoma B, Milne WK (2015 Dec). A systematic review and qualitative analysis to determine quality indicators for health professions education blogs and podcasts. J Grad Med Educ.

[3] Thoma B, Chan TM, Paterson QS (2015). Emergency medicine and critical care blogs and podcasts establishing an international consensus on quality. Ann Emerg Med.

[4] Lin M, Thoma B, Trueger NS (2015). Quality indicators for blogs and podcasts used in medical education: modified Delphi consensus recommendations by an international cohort of health professions educators. Postgrad Med J.

[5] Frequently Asked Questions (2018). Frequently asked questions: emergency medicine. Accreditation council for graduate medical education (ACGME)’s residency review committee for emergency medicine. Published.

[6] Chan TM, Grock A, Paddock M (2016). Examining reliability and validity of an online score (ALiEM AIR) for rating free open access medical education resources. Ann Emerg Med.

[7] Swaminathan A, Morley EJ, Branzetti J (2016). Approved instructional resources series a national initiative to identify quality emergency medicine blog and podcast content for resident education. J Grad Med Educ.

[8] Grock A, Joshi N, Swaminathan A (2016). Blog and podcast watch: neurologic emergencies. West J Emerg Med.

[9] Zaver F, Hansen M, Leibner E (2016). Blog and podcast watch: pediatric emergency medicine. West J Emerg Med.

[10] (2016). CORD testing schedule (2016-2017 Edition). http://www.cordtests.org/.

[11] Thoma B, Sanders JL, Lin M (2015). The social media index: measuring the impact of emergency medicine and critical care websites. West J Emerg Med.

[12] Carpenter CR, Sarli CC, Fowler SA (2013). Best Evidence in Emergency Medicine (BEEM) rater scores correlate with publications' future citations. Acad Emerg Med.

[13] Talan DA, Mower W, Krishnadasan A (2016). Trimethoprim-sulfamethoxazole versus placebo for uncomplicated skin abscess. N Engl J Med.

[14] (2015). 2015 CDC STD treatment guidelines. https://www.cdc.gov/std/tg2015/2015-wall-chart.pdf.

